# Hybrid Experimental–Machine Learning Study on the Mechanical Behavior of Polymer Composite Structures Fabricated via FDM

**DOI:** 10.3390/polym17152012

**Published:** 2025-07-23

**Authors:** Osman Ulkir, Sezgin Ersoy

**Affiliations:** 1 Department of Electric and Energy, Mus Alparslan University, Mus 49210, Türkiye; o.ulkir@alparslan.edu.tr; 2 Ersoy Advanced Research in Mechatronics and Artificial Intelligence, Marmara University, Istanbul 34722, Türkiye; 3 Institute of Microtechnology, Technische Universität Braunschweig, 38124 Braunschweig, Germany

**Keywords:** fused deposition modeling, polymer composite, machine learning, mechanical behavior, additive manufacturing

## Abstract

This study explores the mechanical behavior of polymer and composite specimens fabricated using fused deposition modeling (FDM), focusing on three material configurations: acrylonitrile butadiene styrene (ABS), carbon fiber-reinforced polyphthalamide (PPA/Cf), and a sandwich-structured composite. A systematic experimental plan was developed using the Box–Behnken design (BBD) to investigate the effects of material type (MT), infill pattern (IP), and printing direction (PD) on tensile and flexural strength. Experimental results showed that the PPA/Cf material with a “Cross” IP printed “Flat” yielded the highest mechanical performance, achieving a tensile strength of 75.8 MPa and a flexural strength of 102.3 MPa. In contrast, the lowest values were observed in ABS parts with a “Grid” pattern and “Upright” orientation, recording 37.8 MPa tensile and 49.5 MPa flexural strength. Analysis of variance (ANOVA) results confirmed that all three factors significantly influenced both outputs (*p* < 0.001), with MT being the most dominant factor. Machine learning (ML) algorithms, Bayesian linear regression (BLR), and Gaussian process regression (GPR) were employed to predict mechanical performance. GPR achieved the best overall accuracy with R^2^ = 0.9935 and MAPE = 11.14% for tensile strength and R^2^ = 0.9925 and MAPE = 12.96% for flexural strength. Comparatively, the traditional BBD yielded slightly lower performance with MAPE = 13.02% and R^2^ = 0.9895 for tensile strength. Validation tests conducted on three unseen configurations clearly demonstrated the generalization capability of the models. Based on actual vs. predicted values, the GPR yielded the lowest average prediction errors, with MAPE values of 0.54% for tensile and 0.45% for flexural strength. In comparison, BLR achieved 0.79% and 0.60%, while BBD showed significantly higher errors at 1.76% and 1.32%, respectively.

## 1. Introduction

AM has fundamentally transformed product development by offering unprecedented flexibility in the design and fabrication of complex geometries [1]. Among the various AM techniques, FDM stands out due to its simplicity, cost-effectiveness, and compatibility with a wide range of thermoplastic materials [2]. FDM is extensively used across various industries, including aerospace, automotive, biomedical, and consumer electronics, where rapid prototyping and functional part production are essential [3,4,5]. However, despite its widespread adoption, enhancing the mechanical performance of FDM-printed parts remains a critical challenge, especially for structural applications where reliability and strength are paramount.

The mechanical behavior of FDM components is influenced by multiple interrelated parameters, including material type, infill pattern, printing orientation, layer thickness, and nozzle temperature [6,7]. Polymer-based composite materials are widely studied for various engineering applications, including well-kill operations under high-pressure fracturing conditions [8]. While polymers like ABS are widely used for their ease of processing and dimensional stability [9,10], fiber-reinforced composites, such as PPA/Cf, offer significantly improved stiffness, strength, and thermal resistance. In recent years, hybrid material structures, particularly sandwich composites, have gained attention for their ability to combine the benefits of multiple materials within a single printed part [11,12,13]. These structures aim to balance strength, toughness, and weight, making them ideal for advanced engineering applications. Numerous studies have attempted to optimize FDM process parameters to improve mechanical properties [14,15,16]. Although such studies have demonstrated the effectiveness of both experimental designs and data-driven methods, most existing works are limited to evaluating single material systems or individual mechanical properties, such as tensile strength or surface roughness. There remains a significant gap in the literature regarding the simultaneous analysis and prediction of multiple mechanical properties, particularly in hybrid structures that combine distinct material layers like ABS and PPA/Cf.

Traditional trial-and-error methods and even structured experimental designs often fall short in capturing the nonlinear and complex interactions between FDM process parameters and mechanical responses [17,18]. In response to these limitations, ML has emerged as a transformative tool in the field of smart manufacturing, enabling data-driven insights, real-time process monitoring, and predictive modeling for optimized performance [19,20,21,22]. ML algorithms have shown great promise in reducing experimental costs, accelerating design cycles, and improving product quality by learning from limited datasets in AM. Techniques such as BLR and GPR are especially suitable for small to medium-sized experimental datasets, as they provide not only highly accurate predictions but also uncertainty quantification, which is crucial in engineering design. In the context of FDM, several recent studies have incorporated ML to predict mechanical properties [23,24,25,26,27]. Jain et al. conducted a comprehensive study comparing ML models to predict the flexural behavior of multi-walled carbon nanotube (MWCNT)-reinforced PLA composites fabricated via material extrusion [28]. The research focused on key printing parameters—layer thickness, raster orientation, and feed rate—and their influence on flexural strength, which ranged from 60.618 MPa to 130.935 MPa in tested specimens. Using eight regression algorithms, the study found that the extreme gradient boost (XGBoost) model performed best, achieving a root mean square error (RMSE) of 1.776, a mean absolute error (MAE) of 1.366, and a coefficient of determination (R^2^) of 0.99. Post-test fractography analysis also assessed microstructural integrity. The findings highlight the potential of ML in AM for optimizing process parameters and improving material performance. This approach offers a data-driven pathway to enhance the mechanical properties of 3D-printed nanocomposites. Ulkir et al. investigated the relationship between FDM printing parameters and mechanical properties in carbon fiber-reinforced nylon composites (PA12-CF) using experimental and ML approaches [29]. Their study employed a Taguchi L27 orthogonal array to analyze five critical printing parameters: layer thickness, infill pattern, nozzle temperature, printing speed, and infill density. The results demonstrated that infill density was the most significant factor affecting tensile strength (53.47% contribution), with maximum strength reaching 69.65 MPa at 90% density. For surface roughness, layer thickness showed the highest influence (53.84% contribution), where 100 µm layers produced the best surface finish (9.18 µm). The researchers developed an artificial neural network model that achieved exceptional predictive accuracy (R^2^ > 0.9912, error < 0.41%), outperforming other ML algorithms. These findings underscore the potential of combining statistical analysis with ML to optimize FDM parameters for enhanced mechanical performance in fiber-reinforced composites. The study provides valuable insights for improving the quality and reliability of additively manufactured composite components. Khusheef et al. developed an innovative predictive approach using in-process sensing to enhance property prediction in FDM [24]. By integrating inertial measurement unit sensors and a thermal camera with machine parameters, they analyzed five mechanical properties: surface roughness, tensile strength, elongation at break, micro-hardness, and warpage. A hybrid CNN-LSTM deep learning model processed time-series sensor data encoded as signal images, achieving 99% correlation for tensile strength prediction, though warpage and micro-hardness showed lower accuracy, indicating other influencing factors. The signal imaging technique surpassed traditional feature selection methods, demonstrating its potential to improve AM reliability. This work paves the way for broader industrial adoption of AM by addressing inconsistencies in part quality. While these studies highlight the growing role of ML in AM, they are generally limited to single-material systems, single mechanical properties, or simple geometries. In contrast, the present study distinguishes itself by combining statistically designed experiments (BBD) with ML-based modeling (BLR and GPR) for the simultaneous prediction of tensile and flexural strength in multi-material structures, including a novel sandwich composed of ABS and PPA/Cf. This integrated approach not only enables a deeper understanding of the synergistic effects of material configuration, infill pattern, and printing orientation but also provides a robust and generalizable predictive framework for optimizing mechanical performance in hybrid FDM structures—a topic that remains largely underexplored in the current literature.

This study proposes a hybrid experimental and ML framework to analyze and predict the mechanical behavior of FDM-printed polymer composites. Three material configurations were examined: (i) ABS, (ii) PPA/Cf, and (iii) a novel sandwich structure combining half ABS and half PPA/Cf to balance ductility and stiffness. Using a BBD, the effects of MT, IP, and PD on tensile and flexural strength were systematically investigated. Mechanical tests followed ISO 527 [30] and ASTM D790 [31] standards. In addition to experiments, BLR and GPR models were developed to predict mechanical properties. GPR showed superior accuracy, achieving R^2^ values of 0.9935 and 0.9925 and MAPE values around 11–13% for tensile and flexural strength, respectively. Validation on unseen data demonstrated the GPR’s robustness with an MAPE as low as 0.54% (tensile) and 0.45% (flexural). This study integrates composite design, statistical experimentation, and ML to provide a reliable predictive framework for optimizing FDM printing parameters, enhancing the production of mechanically robust polymer parts.

## 2. Materials and Methods

### 2.1. Materials

In this study, three distinct material configurations were selected to investigate the mechanical performance of FDM-printed polymer composites: ABS, PPA/Cf, and a hybrid sandwich structure composed of both materials. ABS is a widely used thermoplastic in AM due to its good dimensional stability, ease of processing, and balanced mechanical properties [32]. It offers moderate strength and toughness, making it suitable for prototyping and functional applications. PPA/Cf is a high-performance composite filament consisting of a polyphthalamide matrix reinforced with short carbon fibers [33]. The inclusion of carbon fibers significantly enhances the stiffness, strength, and thermal stability of the base material, making it suitable for structural applications requiring high mechanical integrity. PPA/Cf delivers superior performance compared to conventional unfilled polymers, particularly in terms of its tensile and flexural behavior. The filament used in this study was Bambu Lab PPA-Cf Black, a high-performance carbon fiber-reinforced polyphthalamide composite. According to the manufacturer, the material includes approximately 15 wt% chopped carbon fiber, uniformly distributed within the PPA matrix. It exhibits excellent mechanical strength, dimensional stability, and thermal resistance, with a flexural modulus of approximately 9860 MPa and flexural strength of 208 MPa in the dry state. The filament also maintains performance under humid and high-temperature conditions, making it suitable for demanding engineering applications. The third material configuration used in this study is a sandwich-structured composite, which strategically combines the properties of both ABS and PPA/Cf within a single specimen. For these hybrid samples, the lower half of the specimen was printed using ABS, while the upper half was printed with PPA/Cf. This arrangement was designed to leverage the ductility and impact resistance of ABS along with the high stiffness and strength of PPA/Cf, aiming to achieve a mechanically balanced and material-efficient structure. The sandwich configuration represents a novel approach to hybrid multi-material FDM, offering potential advantages in customized mechanical performance for advanced engineering applications. All filaments used in this study had a diameter of 1.75 mm and were processed under optimal storage conditions to ensure consistent material quality. The selection of these three material configurations allowed for a comprehensive assessment of how MT affects the tensile and flexural strength of FDM-printed parts under varying process conditions.

### 2.2. Fused Deposition Modeling Process

**Figure 1:**
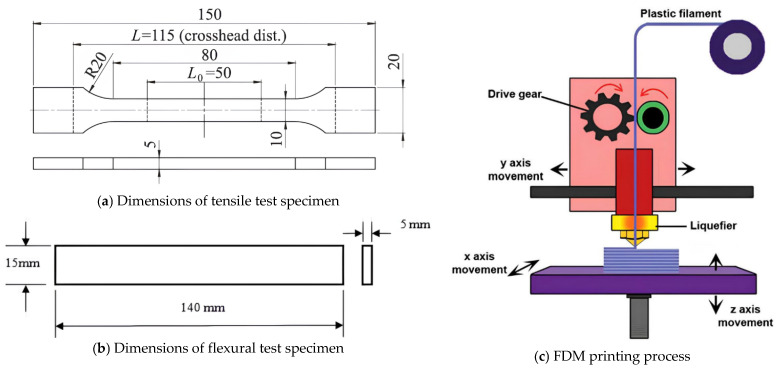
Technical drawing of the tensile and flexural test specimen and FDM process.

All test specimens in this study were fabricated using the FDM technique with a Zaxe Z1 Plus desktop 3D printer (Zaxe Inc., Istanbul, Turkey). This printer features a direct-drive extrusion system, a heated build platform, and a fully enclosed chamber, which enables high-quality printing of both standard thermoplastics and fiber-reinforced composites. In the FDM process, thermoplastic filaments are fed into a heated nozzle, melted, and deposited layer by layer onto the build platform following predefined tool paths. This layer-by-layer deposition allows for the creation of complex geometries and multi-material parts with controlled internal structures. A schematic representation of the FDM process is provided in Figure 1c. The tensile test specimens were designed in accordance with the ISO 527 Type 1A standard, which specifies dog-bone-shaped samples with defined gauge dimensions to ensure consistent mechanical testing. The flexural test specimens followed the ASTM D790 standard, which defines rectangular bars suitable for three-point bending tests. The technical drawings of both specimen types are illustrated in Figure 1a for tensile samples and Figure 1b for flexural samples, respectively. Three types of material configurations were used: (i) ABS, a widely utilized thermoplastic offering balanced mechanical properties and ease of processing; (ii) PPA/Cf, a carbon fiber-reinforced polyphthalamide composite with superior strength and stiffness; and (iii) a sandwich-structured composite, in which the lower half of the specimen was printed with PPA/Cf and the upper half with ABS. This hybrid structure was achieved by pausing the print at the mid-height layer, manually retracting the PPA/Cf filament, and then loading the ABS filament into the same extruder. After ensuring proper extrusion and nozzle priming, the print was resumed to complete the upper half of the specimen with ABS. While this manual switching process enabled the fabrication of a continuous two-material part without the need for multi-nozzle hardware, it introduced potential challenges related to interlayer adhesion between the dissimilar materials. To mitigate this, a short purge routine and temperature stabilization period were applied before resuming the print, ensuring proper bonding at the material interface. Visual inspection and mechanical testing results indicated no significant delamination or defects at the transition zone, suggesting that the applied procedure was effective in maintaining acceptable print quality and structural integrity.

**Figure 2:**
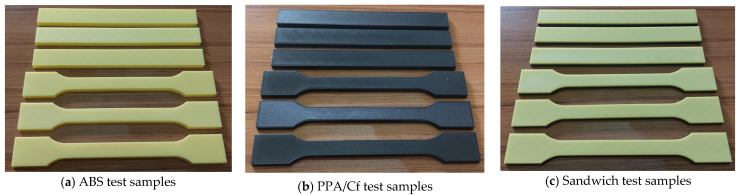
The fabricated ABS, PPA/Cf, and sandwich test samples via FDM.

The experimental plan was constructed using a BBD, which involved 15 unique parameter combinations based on three independent variables: MT, IP, and PD (Table 2). For each combination, three identical specimens were fabricated to ensure statistical reliability, resulting in a total of 45 tensile and 45 flexural test samples. The printed parts exhibited high dimensional fidelity and surface quality across all configurations. Figure 2 shows examples of the fabricated specimens for each material group—ABS, PPA/Cf, and sandwich composites—highlighting the visual and structural distinction between material types. These samples were subsequently subjected to mechanical testing in accordance with the relevant standards.

### 2.3. Design of Experiments

The mechanical performance of printed parts is highly sensitive to various process parameters in FDM. Factors such as material type, infill pattern, printing orientation, layer thickness, nozzle temperature, and printing speed influence not only the dimensional accuracy and surface quality of the parts but also their tensile strength, flexural strength, and overall structural integrity. Among these, MT, IP, and PD are particularly critical, as they directly affect the bonding between layers, fiber alignment (in composite materials), and internal stress distribution during mechanical loading. Specifically, MT determines the intrinsic mechanical properties of the printed part, such as stiffness, ductility, and fiber reinforcement characteristics. For instance, fiber-filled composites behave differently from pure polymers in terms of stress transfer and crack propagation. IP governs the internal geometry of the part, which plays a crucial role in distributing mechanical loads and mitigating stress concentrations. Certain patterns can lead to improved load paths and more uniform strain distribution across the structure. PD is directly related to the orientation of deposited layers with respect to the load direction. Since FDM parts often exhibit anisotropic behavior, the orientation of the print layers significantly influences interlayer bonding strength and failure mechanisms under tensile or bending loads. Therefore, these three parameters were selected for focused investigation in this study due to their direct and combined impact on the mechanical integrity of FDM-printed components. Although other parameters like layer thickness, nozzle temperature, and printing speed are also known to influence mechanical behavior, they were intentionally held constant throughout this study to isolate the effects of the structural and material parameters being investigated. Including additional parameters would require a significantly larger number of experimental runs, especially under the BBD framework. To systematically assess the effects of these parameters and their interactions, a BBD approach was employed. BBD is a response surface methodology (RSM) technique widely used for optimizing multi-variable systems with a minimal number of experiments [34,35]. Unlike full factorial designs, BBD avoids extreme-level combinations and focuses on midpoint and edge-center points of the design space, making it more efficient and practical for experimental studies. This design is particularly advantageous in AM, where certain combinations of parameters may result in print failures or inconsistent quality. Three independent variables were selected as the key factors for this study: MT, IP, and PD. The MT included three levels: ABS, PPA/Cf, and a hybrid sandwich structure combining both materials. The IP was varied as Triangles, Grid, and Cross, while the PD was considered in three orientations: Flat, On-edge, and Upright. Patterns were chosen based on their widespread use in FDM, predictable load distribution, and compatibility with most slicing software. Additionally, they offer varying directional stiffness characteristics, making them suitable for comparative mechanical analysis under tensile loading. While other patterns, such as honeycomb or gyroid, are also popular due to their energy absorption, lightweight properties, and isotropic strength distribution, they were excluded in this study to limit the scope and maintain a manageable experimental design. The levels and symbolic representations of each parameter are summarized in Table 1.

Based on the BBD methodology, a total of 15 experimental runs were generated, each representing a unique combination of the selected factors (Table 2). For each configuration, three identical specimens were fabricated for both tensile and flexural testing, resulting in 45 tensile and 45 flexural samples in total. The complete list of experimental configurations is provided in Table 2. It is important to note that some parameter combinations appear more than once. This is a deliberate characteristic of the BBD, which includes replicated center points to enable the estimation of experimental error and to improve model robustness. These repeated runs help evaluate the reproducibility of the process and provide a statistical basis for assessing the lack of fit in the regression model. In this way, BBD not only identifies significant main and interaction effects but also supports the development of accurate and reliable predictive models. The BBD was selected over other response surface methodologies, such as central composite design (CCD), due to several practical and statistical considerations aligned with the objectives of this study. First, BBD is highly efficient for three-level factorial designs and requires fewer experimental runs than the CCD when the number of variables increases, making it more cost-effective, especially in material testing applications where each print and test can be time-consuming and resource-intensive. Second, unlike CCD, BBD does not include extreme combinations (i.e., corner points) of all factors at their lowest or highest levels simultaneously, which helps avoid impractical or potentially damaging fabrication conditions in FDM processes. This is particularly important in AM, where extreme settings may lead to print failure or non-representative results. Furthermore, the BBD provides good model-fitting capability for second-order polynomial regression, which is essential for capturing nonlinear relationships among process parameters. This structured experimental design aims to uncover the individual and combined effects of key FDM parameters on mechanical performance and to lay the foundation for data-driven optimization through regression modeling and ML techniques. The use of BBD ensures a balanced and statistically robust framework to support both traditional response surface analysis and the integration of ML-based predictive modeling.

### 2.4. Box–Behnken Design

The BBD is a widely used experimental design method within the framework of RSM. It is specifically developed for modeling and analyzing problems in which a response of interest is influenced by several independent variables, and the objective is to optimize this response. Unlike full factorial or CCD, BBD requires fewer experimental runs, making it more efficient and cost-effective, particularly when dealing with three or more variables [36]. A key characteristic of BBD is that it avoids combinations where all factors are at their extreme levels simultaneously, thereby reducing the likelihood of conducting experiments under potentially unsafe or impractical parameter settings. Moreover, BBD includes replicated center points to estimate pure experimental error, enabling statistical validation of the model through ANOVA [37]. In this study, the BBD was employed to investigate the effects of three key FDM parameters—MT, IP, and PD—on the tensile and flexural strength of 3D-printed parts. Each factor was evaluated at three levels, and the design generated experimental combinations. The structured nature of BBD not only allows for the estimation of main effects but also enables the analysis of two-way interactions and potential quadratic effects. This makes it particularly suited to capturing the nonlinear behavior commonly observed in AM processes. The experimental results obtained from these BBD-configured trials provided the foundation for developing regression-based and ML models aimed at predicting mechanical performance with high accuracy.

### 2.5. Experimental Tests

The mechanical performance of the FDM-printed specimens was evaluated using two standard tests: tensile and flexural (bending) tests. These tests were performed under controlled laboratory conditions to ensure accuracy, repeatability, and adherence to international standards. The tensile tests were performed in accordance with the ISO 527-2 standard, which specifies the testing procedure for plastics under uniaxial tensile loading. Specimens used for this test were manufactured in the Type 1A dog-bone geometry, as shown previously in Figure 1a. The tests were conducted using a universal testing machine (UTM) equipped with a 10 kN load cell and pneumatic grips to prevent slippage during elongation. The crosshead speed was set to 5 mm/min, as specified by the ISO standard for the selected specimen dimensions and material type. During the test, real-time data for load and displacement were recorded, and tensile strength (MPa) was calculated by dividing the maximum load by the cross-sectional area of the narrow section of the specimen. The flexural tests were performed according to the ASTM D790 standard, which outlines the procedure for three-point bending tests on plastic materials. Rectangular bar-shaped specimens, as depicted in Figure 1b, were placed on two supports with a span length of 64 mm, and a central loading nose applied force at a constant crosshead speed of 2 mm/min. The flexural strength (MPa) was determined using the formula provided in the ASTM D790 standard, based on the maximum load applied before fracture or yielding, the support span, and the specimen dimensions. For each experimental configuration defined by the BBD, three identical samples were tested to ensure statistical reliability. The average values of tensile and flexural strength were used as the response variables in subsequent regression and ML. All samples were visually inspected before and after testing to identify possible defects such as delamination, warping, or incomplete fusion, which could affect mechanical performance. The testing process revealed consistent mechanical behavior across repeated specimens, confirming the reliability of both the fabrication and testing procedures. The collected experimental data served as the foundation for developing the predictive models discussed in the following sections.

### 2.6. Machine Learning Algorithms

ML has emerged as a powerful tool for modeling and predicting complex, nonlinear relationships in engineering applications [38,39], particularly in AM, where multiple interacting parameters influence part performance [40,41]. In this study, two supervised learning algorithms were employed to develop predictive models for tensile strength and flexural strength based on the experimental data obtained from the BBD: BLR and GPR. Both algorithms are well-suited for small to medium-sized datasets, offering robust performance and uncertainty quantification, which are essential for design validation and risk assessment in engineering contexts. BLR is a probabilistic extension of classical linear regression that incorporates prior distributions over the model parameters [42,43]. This approach not only provides point estimates but also yields a distribution of possible outcomes, enabling confidence intervals around predictions. BLR is particularly advantageous when dealing with limited datasets, as it effectively prevents overfitting by regularizing the model based on prior knowledge. GPR is a non-parametric, kernel-based algorithm that models the underlying function as a distribution over possible functions [44,45]. GPR is highly flexible and capable of capturing complex, nonlinear relationships between input variables and outputs. Additionally, it provides predictive uncertainty for each estimate, making it a valuable tool for high-stakes applications where reliability is critical. In this study, both models were trained using the experimental data and evaluated based on performance metrics such as R^2^ and MAPE, offering a comparative assessment of their predictive capabilities.

## 3. Results and Discussion

### 3.1. Box–Behnken Design Results

The experimental results obtained from the BBD are presented in Table 3, which summarizes the tensile and flexural strength values corresponding to each of the 15 parameter combinations. These results provide valuable insights into how the selected printing parameters influence the mechanical behavior of FDM-printed parts. Specimens fabricated with PPA/Cf consistently exhibited the highest mechanical performance across all configurations. The highest tensile strength (75.8 MPa) and flexural strength (102.3 MPa) were recorded in Run 3, where the PPA/Cf material was used with “Cross” infill and “Flat” direction. This indicates that the synergistic effect of optimized fiber alignment and infill geometry plays a crucial role in enhancing load-bearing capacity. In contrast, ABS samples showed the lowest mechanical properties, with the minimum tensile strength (37.8 MPa) and flexural strength (49.5 MPa) observed in Run 11 (“Grid” infill, “Upright” direction), highlighting the relatively lower stiffness and interlayer adhesion of ABS compared to fiber-reinforced composites. The sandwich structures, which combine the ductility of ABS and the stiffness of PPA/Cf, demonstrated intermediate performance between the two pure materials. For instance, in Run 5 (“Grid” infill, “Flat” direction), the sandwich specimen achieved a tensile strength of 62.1 MPa and a flexural strength of 84.1 MPa, outperforming pure ABS while remaining below the best-performing PPA/Cf samples. This confirms the effectiveness of the hybrid configuration in balancing strength and material economy. The sandwich structure, which positions carbon fiber-reinforced PPA at the outer skins and unreinforced material at the core, exhibits intermediate mechanical performance compared to full-carbon fiber or full-unreinforced configurations. This balanced behavior offers a practical compromise between strength, weight, and material cost. Such a configuration is particularly advantageous in applications where moderate mechanical strength, reduced weight, and material efficiency are all critical design requirements. This structure may be highly suitable for automotive interior components, drone or UAV airframes, protective casings, or tooling fixtures, where stiffness and dimensional stability are needed, but full reinforcement may be unnecessary or economically inefficient. Moreover, the sandwich layout may contribute to vibration damping and thermal insulation, making it a compelling choice for multi-functional engineering parts. These practical implications highlight the potential of the sandwich configuration in lightweight structural design.

Another critical factor influencing mechanical outcomes was the PD. Specimens printed in the “Flat” or “On-edge” orientations generally yielded higher tensile and flexural strengths compared to those printed in the “Upright” direction. This trend can be attributed to improved interlayer bonding and better alignment of load paths with the direction of applied force in Flat/On-edge orientations. For example, comparing Runs 2 (ABS, Grid, Flat) and 11 (ABS, Grid, Upright), a significant drop in both tensile and flexural strength is evident when the orientation changes from Flat to Upright. Lastly, the effect of IP also manifested noticeably, particularly for PPA/Cf and sandwich specimens. The “Cross” pattern often resulted in slightly higher mechanical strength compared to “Grid” or “Triangles”, likely due to its more uniform stress distribution and enhanced internal connectivity. The influence on IP appears to be secondary to that of MT and PD. The BBD results clearly indicate that MT is the dominant factor affecting mechanical performance, followed by PD and IP. These findings form the basis for subsequent regression modeling and ML analysis, allowing for data-driven prediction and optimization of part strength in FDM processes.

### 3.2. Interaction Effect Plots

**Figure 3:**
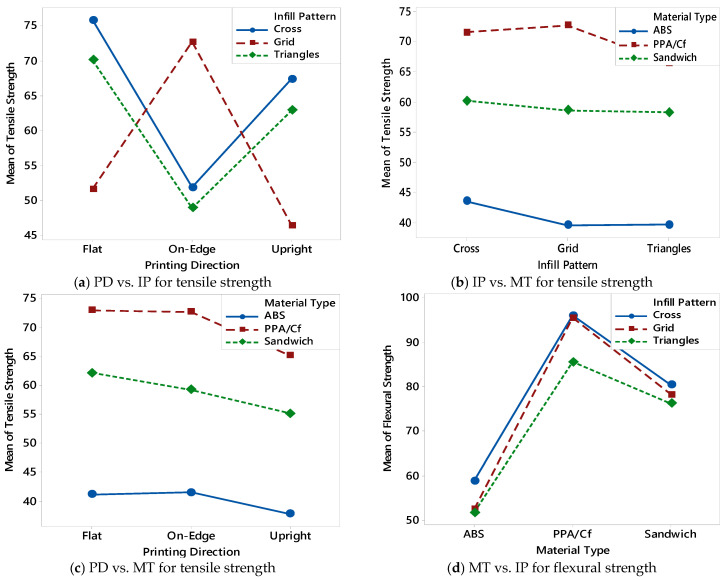

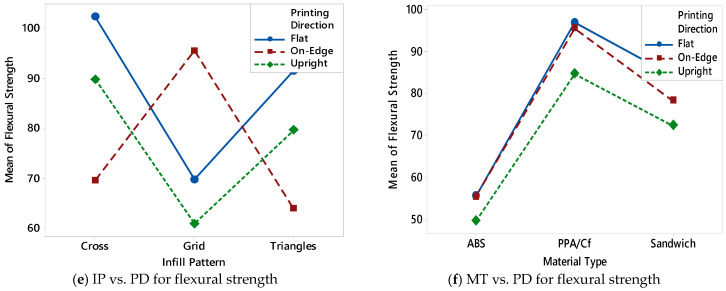
Interaction charts between printing parameters based on tensile and flexural strength results.

Interaction effect plots were generated using the experimental results from the BBD to better understand the combined effects of printing parameters on mechanical performance. These interaction charts, shown in Figure 3a–f, illustrate how two parameters interactively influence the tensile and flexural strength of FDM-printed specimens. Such visualizations are crucial for identifying nonlinear trends and synergistic or antagonistic relationships between variables. In Figure 3a, the interaction between PD and IP is presented. The results show that the “Flat” PD results in the highest strengths across most patterns, especially for the “Cross” pattern. However, when the direction changes to “On-edge” or “Upright”, performance decreases significantly for certain patterns like “Grid”, indicating a strong dependency between build orientation and internal geometry. Figure 3b displays the interaction between IP and MT. PPA/Cf consistently outperforms ABS and sandwich materials across all IPs. The “Grid” pattern yields relatively better results for PPA/Cf, while “Cross” and “Triangles” perform similarly for sandwich composites. ABS shows the lowest strength values, with minor variation across patterns, suggesting that the effect of infill pattern on ABS is less pronounced than on stiffer materials. The interaction between PD and MT is illustrated in Figure 3c. Across all orientations, PPA/Cf exhibits the highest strength, especially in the “Flat” and “On-edge” directions. However, a noticeable drop is observed in the “Upright” direction for all materials. This confirms the well-known limitation of FDM when printing in the vertical direction, where interlayer adhesion is weaker, leading to reduced tensile and flexural strength. In Figure 3d, the interaction plot for MT versus IP is revisited to highlight potential nonlinear relationships. The “Cross” pattern again emerges as the most favorable infill structure for both PPA/Cf and sandwich materials, while ABS remains relatively unaffected. This reinforces the idea that internal geometry plays a more critical role in high-performance or fiber-reinforced materials. Figure 3e further explores the interaction between IP and PD. The results are consistent with earlier findings: “Flat” and “On-edge” directions generally yield better strength values, particularly when combined with “Cross” or “Grid” patterns. The “Upright” direction shows poor performance regardless of infill type, emphasizing its mechanical disadvantage. Finally, Figure 3f examines the interaction between MT and PD. PPA/Cf again shows superior strength in the “Flat” direction, followed by “On-edge”, while “Upright” causes the greatest reduction in performance. Sandwich composites follow a similar trend but with slightly lower values, and ABS consistently ranks lowest, especially in the “Upright” direction. These results confirm that both material stiffness and layer bonding orientation are dominant factors in determining the mechanical integrity of FDM parts. The interaction plots in Figure 3 reveal that the combined influence of printing parameters is often more significant than their individual effects. This highlights the necessity of using multi-factorial experimental designs like BBD and supports the implementation of data-driven models for accurate prediction and optimization of mechanical performance in FDM processes.

**Figure 4:**
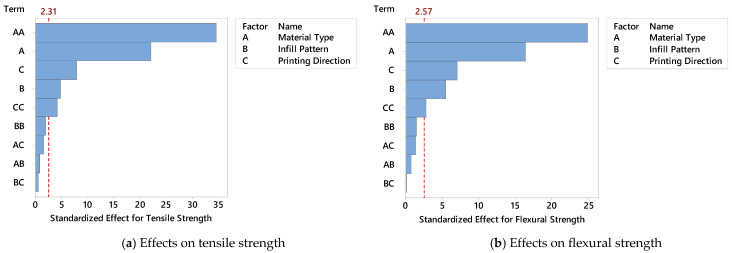
The Pareto chart of the standardized effects for the BBD.

Pareto charts of standardized effects, created using the results from the BBD, provided a quantitative view of how each printing parameter influenced mechanical performance. These charts—shown in Figure 4a for tensile strength and Figure 4b for flexural strength—visualize the magnitude and statistical significance of each factor’s effect, ranked from most to least influential. The vertical red dashed lines indicate the critical t-value thresholds (2.31 for tensile and 2.57 for flexural strength), above which the effects are considered statistically significant at a 95% confidence level. MT (Factor A) has by far the most dominant effect on tensile strength, exceeding the significance threshold by a wide margin (Figure 4a). This result aligns with previous findings, where specimens printed with PPA/Cf consistently outperformed those made from ABS or sandwich composites. The IP (Factor B) also shows a statistically significant but smaller effect, indicating that internal geometry plays a secondary yet meaningful role in enhancing tensile performance. PD (Factor C) falls below the significance threshold, suggesting that while orientation has some influence—particularly on interlayer bonding—its effect on tensile strength is comparatively limited when material and infill are optimized. Similarly, Figure 4b shows the Pareto chart for flexural strength, and the trends are consistent. MT remains the most influential factor, followed by IP and PD, both of which again fall below the critical threshold. The slightly higher critical value (2.57) for flexural strength may explain why fewer parameters reach significance, but the overall hierarchy remains the same. These Pareto analyses confirm that MT is the single most critical factor affecting both tensile and flexural performance of FDM-printed parts. While IP can fine-tune the performance, especially in composite or hybrid structures, printing direction alone does not yield statistically significant changes under the tested conditions. These insights are essential for guiding parameter selection during the design phase, and they also validate the structure of the predictive models developed in the following sections.

### 3.3. Analysis of Variance Results

The statistical significance of the selected process parameters on both tensile and flexural strength was evaluated using ANOVA. The results are summarized in Table 4, which presents the degrees of freedom (DF), adjusted sums of squares (Adj SS), mean squares (Adj MS), F-values, and corresponding *p*-values for each factor. For tensile strength, the ANOVA results indicate that all three parameters have statistically significant effects at the 95% confidence level. MT is the most influential factor, with a very high F-value of 201.44 and a *p*-value of 0.000, confirming its dominant role in determining tensile performance. PD also shows a highly significant effect (F = 64.50, *p* = 0.000), highlighting the importance of layer orientation and interlayer bonding in tensile loading. IP, while less dominant, still contributes significantly (F = 24.87, *p* = 0.003), suggesting that internal geometry affects stress distribution and crack propagation paths. A similar trend is observed for flexural strength. Again, MT has the highest impact (F = 102.23, *p* = 0.000), followed by PD (F = 31.13, *p* = 0.000) and IP (F = 17.07, *p* = 0.001). These values confirm that the bending behavior of FDM parts is also strongly dependent on the stiffness of the material and the orientation of the printed layers. The *p*-values for all three parameters are below 0.005, indicating a high level of statistical significance. The lack-of-fit test was also included to assess the adequacy of the regression model. For tensile strength, the lack-of-fit F-value is 29.95 with a *p*-value of 0.033, indicating a statistically significant lack of fit. This suggests that while the model captures the general trend well, there may be some non-linearities or interactions not fully accounted for by the current model. Potential sources of this lack of fit may include nonlinear interactions between process parameters (e.g., material type and infill pattern), as well as microstructural variability introduced during manual filament switching in the sandwich-structured specimens. Furthermore, higher-order effects or unmodeled dependencies may also contribute to the unexplained variance. Although the model provides reasonable predictive performance, these findings highlight the need for more complex modeling approaches—such as including additional interaction terms or applying nonlinear regression techniques—in future studies to reduce the lack of fit and improve model fidelity. In contrast, for flexural strength, the *p*-value for lack of fit is 0.095, which is above the 0.05 threshold, indicating that the model fits the data reasonably well for bending behavior. The ANOVA findings confirm that all three printing parameters have a statistically significant influence on mechanical properties. Among them, MT consistently exhibits the greatest impact, followed by PD and IP.

**Figure 5:**
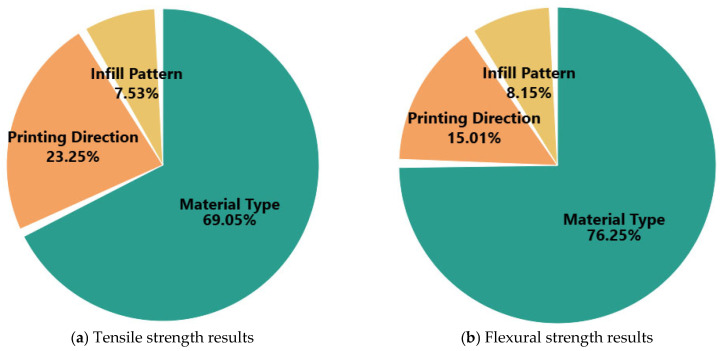
Contribution of each printing parameter to the tensile and flexural strength of the parts.

The percentage contributions of MT, IP, and PD to the mechanical performance of FDM-printed parts were calculated based on their Adj SS values obtained from the ANOVA results, allowing further quantification of each parameter’s relative impact. The results are visualized in Figure 5a for tensile strength and in Figure 5b for flexural strength. As depicted in Figure 5a, for tensile strength, MT is the dominant factor, contributing 69.05% of the total variation in the response. This reinforces the earlier statistical findings that the intrinsic properties of the materials, such as stiffness, fiber reinforcement, and interlayer bonding, play a critical role in load-bearing capacity. PD contributes 23.25%, indicating that layer orientation and the direction of applied stress significantly affect tensile behavior, particularly due to anisotropic properties inherent in FDM processes. IP, while statistically significant, accounts for only 7.53% of the variation, suggesting that although internal geometry influences stress distribution, its effect is relatively minor compared to the other two parameters. In the case of flexural strength, shown in Figure 5b, the contribution of MT is even more pronounced, rising to 76.25%. This suggests that bending resistance is even more sensitive to material stiffness and structural composition than tensile strength. PD contributes 15.01%, while IP accounts for 8.15%, showing a similar trend of decreasing influence. The relatively smaller impact of printing direction in bending (compared to tensile) could be attributed to the fact that bending loads are more evenly distributed along the part’s cross-section, partially mitigating the anisotropic effects. These percentage-based contribution analyses confirm that MT is by far the most critical parameter in determining both tensile and flexural performance. While PD plays a supportive yet significant role, especially in tensile loading, IP serves as a fine-tuning parameter that can help optimize performance when combined with appropriate materials and orientations. The outcome of this analysis supports the rationale for prioritizing material selection in both design and predictive modeling, and it further validates the regression and ML models developed in the subsequent sections.

### 3.4. Regression Model Results

Predictive models for estimating the mechanical properties of FDM-printed parts were developed using multiple regression analysis based on experimental data from the BBD. The models aim to correlate the effects of MT, IP, and PD with the resulting tensile and flexural strength values. Quadratic regression equations were derived to account for potential non-linearities and interaction effects. The resulting regression equations are as follows:

(1) $${\mathrm{Tensile}\;\mathrm{Strength}}_{\mathrm{BBD}}\;=\;72.733\;+\;9.20\mathrm{MT}\;+\;2\mathrm{IP}-3.25\mathrm{PD}-21.167{\mathrm{MT}}^{2}-1.167{\mathrm{IP}}^{2}-2.517{\mathrm{PD}}^{2}\;{\mathrm{Flexural}\;\mathrm{Strength}}_{\mathrm{BBD}}\;=\;95.53\;+\;12.188\mathrm{MT}\;+\;4.05\mathrm{IP}-5.263\mathrm{PD}-27.10{\mathrm{MT}}^{2}-1.63{\mathrm{IP}}^{2}-3.10{\mathrm{PD}}^{2}$$

**Figure 6:**
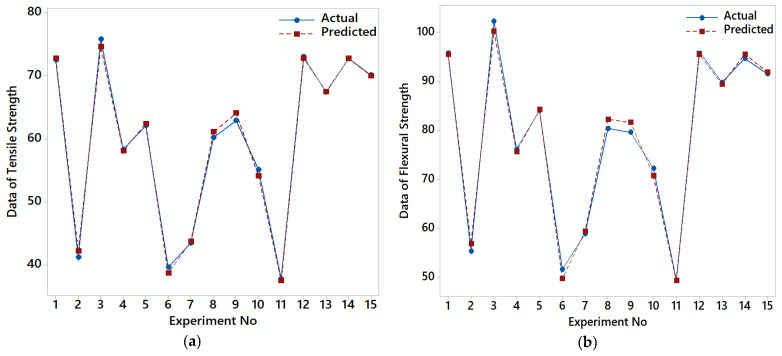

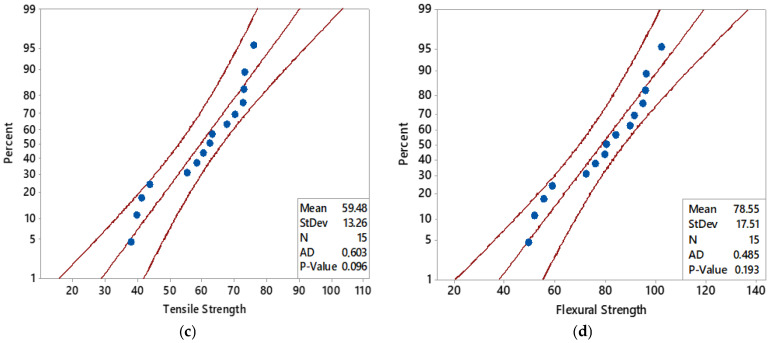
Graphs of the tensile–flexural strength regression analysis results for the BBD. (**a**) Timescale graph for tensile strength; (**b**) Timescale graph for flexural strength; (**c**) Normal probability plot of the tensile strength; (**d**) Normal probability plot of the flexural strength.

These equations show that MT has the highest linear and quadratic influence on both tensile and flexural strength, consistent with the findings from ANOVA and contribution analyses. The negative quadratic coefficients indicate diminishing returns or possible optimal levels for each parameter. The accuracy and validity of the regression models were assessed using R^2^ and diagnostic plots. The R^2^ value for tensile strength was found to be 0.9895, while for flexural strength, it was 0.9885. These high R^2^ values indicate that the models explain more than 98% of the variability in the experimental data, demonstrating excellent fitting performance. The timescale plots compare the actual and predicted values across 15 experimental runs (Figure 6a,b). The predicted data closely follows the actual measurements, with minimal deviation, further verifying the robustness of the regression models. The normal probability plots in Figure 6c for tensile strength and Figure 6d for flexural strength illustrate that the residuals approximately follow a normal distribution. The *p*-values obtained from Anderson–Darling tests are 0.096 and 0.193 for tensile and flexural strength, respectively—both above the 0.05 threshold—indicating no significant deviation from normality and thus validating the assumptions of the regression analysis. The regression models developed in this study offer high predictive accuracy and satisfy the assumptions of linear regression. These models serve as reliable tools for estimating mechanical performance based on key FDM process parameters and form the foundation for more advanced predictive techniques, such as ML algorithms, presented in the following section.

In addition to prediction accuracy, the computational efficiency of the two models is also important for practical implementation in AM workflows. GPR, while highly flexible and capable of modeling complex, nonlinear relationships, is computationally intensive, especially as the training dataset grows. Its training complexity scales cubically with the number of observations (O(n^3^)), which can limit its scalability for large datasets or real-time applications. In contrast, BLR offers significantly greater computational efficiency due to its closed-form posterior inference and simpler model structure. This enables faster training and prediction times, making it more suitable for real-time decision-making or integration into AM control systems where computational resources may be limited. Therefore, while GPR may provide slightly higher accuracy in modeling complex behaviors, BLR presents a favorable trade-off in scenarios where speed and resource efficiency are critical.

### 3.5. Machine Learning Algorithm Results

In recent years, ML has become an indispensable tool in the field of AM, especially for process optimization and mechanical property prediction. The highly nonlinear and multi-parametric nature of FDM makes it difficult to capture complex interactions using classical statistical models alone. ML algorithms offer a data-driven approach that can learn from experimental data and generate highly accurate predictive models, even with relatively small datasets. In this study, two supervised learning algorithms—GPR and BLR—were implemented to predict the tensile and flexural strength of 3D-printed parts based on the input parameters: MT, IP, and PD. These algorithms were selected due to their suitability for small datasets and their ability to provide probabilistic outputs, including prediction intervals. The ML models were developed and trained using the Regression Learner App in MATLAB. The dataset obtained from the BBD was first normalized and then split into training and validation sets using k-fold cross-validation (k = 5) to ensure generalizability. GPR was implemented with an exponential kernel function, while BLR used a Bayesian regularization approach to prevent overfitting. The exponential kernel was preferred over more commonly used kernels such as the squared exponential (RBF) because it is better suited for capturing less smooth, potentially abrupt variations in the response due to discrete changes in FDM process parameters. Unlike the RBF kernel, which assumes smooth and infinitely differentiable functions, the exponential kernel can accommodate localized, nonlinear effects that are more representative of real-world material behavior in AM. Preliminary cross-validation results showed that the exponential kernel yielded lower prediction error and better generalization on validation data, further justifying its selection.

A systematic hyperparameter optimization process was applied for both GPR and BLR to ensure the robustness, accuracy, and generalizability of the ML models. For GPR, a grid search strategy was employed to evaluate multiple kernel functions and their associated hyperparameters. The tested kernels included Squared Exponential (RBF), Matern 5/2, Rational Quadratic, and Exponential. Among these, the rational quadratic kernel yielded the best predictive performance. Key hyperparameters were optimized within the following ranges: length scale (l) from 0.1 to 5.0 (step size: 0.1), noise level (α) from 1 × 10^−6^ to 1 × 10^−2^ (logarithmic scale), and the scale mixture parameter from 0.1 to 10.0. The optimal configuration was determined as follows: Rational Quadratic kernel with a length scale of 1.2, noise level of 1 × 10^−4^, and a scale mixture parameter of 2.5. Model selection was based on 10-fold cross-validation using R^2^, RMSE, and MAE as performance metrics. For BLR, although the model has fewer tunable parameters, we optimized the prior variance and noise precision using a Type-II maximum likelihood estimation approach. The prior variance (σ^2^_prior) was searched within the range of 0.01 to 10.0, and the noise variance (σ^2^_noise) within 1 × 10^−4^ to 1.0. The best results were obtained with σ^2^_prior = 1.0 and σ^2^_noise = 0.01. These configurations were found to significantly improve the model’s predictive ability and stability. All optimization procedures were carried out using the MATLAB Regression Learner Toolbox and complementary Python scripts utilizing the scikit-learn and GPy libraries. The hyperparameter tuning process was critical in achieving the high prediction accuracy (R^2^ > 0.99) reported in this study. Moreover, providing this level of detail enhances the transparency, reproducibility, and methodological rigor of the research and ensures that the proposed modeling approach can be confidently applied to similar datasets and applications in AM.

**Figure 7:**
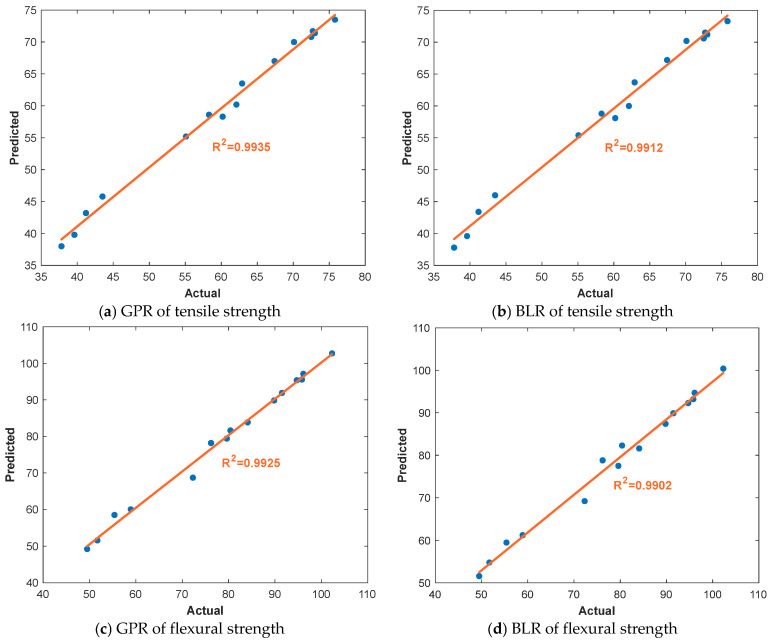
ML results for the tensile and flexural strength of the parts.

Model performance was evaluated based on R^2^ and the visual comparison of predicted versus actual values. The results of the ML models are shown in Figure 7. For tensile strength, the GPR model achieved an R^2^ of 0.9935 (Figure 7a), while the BLR model followed closely with an R^2^ of 0.9912 (Figure 7b). These results indicate that both models successfully captured the underlying relationships between the input parameters and the resulting tensile strength, with GPR offering slightly higher accuracy. Similarly, for flexural strength, the GPR model yielded an R^2^ of 0.9925 (Figure 7c), and the BLR model produced an R^2^ of 0.9902 (Figure 7d). Again, both models demonstrated excellent predictive capability, with GPR slightly outperforming BLR in terms of precision. The data closely follows the 45-degree line in all plots, indicating strong agreement between actual and predicted values. These findings confirm that ML models, particularly GPR, are highly effective in predicting mechanical properties in FDM processes. They not only complement traditional regression models but also offer added flexibility and generalization for unseen data. The successful implementation of ML in this study highlights its potential for real-time prediction, quality control, and process optimization in next-generation AM workflows.

An error performance analysis was conducted to evaluate the predictive performance and generalization ability of the ML models used in this study. The results of the GPR and BLR models were compared with the traditional BBD-based regression model using standard error metrics, including MSE, MAE, RMSE, MAPE, R^2^, and Pearson correlation coefficient (r). The results are presented in Table 5 for tensile strength and Table 6 for flexural strength. The GPR algorithm demonstrated superior accuracy in predicting tensile strength, with the lowest MSE (0.7933), MAE (2.7021), and RMSE (0.8908) values among all models (Table 5). It also achieved the lowest MAPE of 11.135%, indicating minimal deviation from actual values. Additionally, the R^2^ value of 0.9935 and correlation coefficient of 0.9967 confirm the excellent predictive capability and strong linear correlation between predicted and actual values. The BLR model also performed well, with slightly higher error values than GPR but still outperforming the BBD regression model. With an R^2^ of 0.9912 and MAPE of 12.079%, BLR proves to be a reliable and interpretable ML method for modeling nonlinear relationships in FDM processes. In comparison, although the BBD-based regression model also showed high accuracy (R^2^ = 0.9895, MAPE = 13.025%), it lagged slightly behind the ML models, particularly in terms of generalization and error minimization. This highlights the advantage of using ML techniques for capturing the complex and nonlinear interactions between FDM process parameters.

A similar trend was observed in the prediction of flexural strength (Table 6). The GPR model again yielded the best results with the lowest MSE (1.1525), MAE (3.4219), and RMSE (1.0739) values, along with an MAPE of 12.958%. The model achieved an R^2^ of 0.9925 and an r of 0.9968, indicating an almost perfect fit to the experimental data. The BLR model followed closely, with an R^2^ of 0.9902 and MAPE of 13.692%, confirming its effectiveness in modeling flexural behavior. While the BBD regression model also performed adequately (R^2^ = 0.9885, MAPE = 14.102%), its higher error values suggest that it may not capture all nonlinearities and parameter interactions as effectively as the ML models.

### 3.6. Validation Test Results of Prediction Algorithms

The generalization capability and real-world applicability of the developed models were assessed through a validation test using new experimental samples excluded from the training phase. This test aimed to compare the prediction accuracy of the BBD regression model with two ML algorithms, GPR and BLR, for both tensile and flexural strength outputs. The actual and predicted values for each model are presented in Table 7. For tensile strength, the MAPE was calculated as 1.76% for the BBD model, 0.79% for BLR, and the lowest value of 0.54% for the GPR model. Similarly, in the flexural strength predictions, GPR again outperformed with an MAPE of 0.45%, followed by BLR at 0.60% and BBD with 1.32%. These results clearly indicate that both ML models, particularly GPR, offer significantly higher accuracy and generalization performance than the traditional regression approach. The GPR algorithm consistently produced the lowest prediction errors in all validation cases, demonstrating its ability to effectively model the nonlinear and complex relationships among FDM printing parameters. Therefore, GPR stands out as a highly reliable and precise tool for predicting mechanical performance in AM applications.

### 3.7. Discussion

The findings of this study demonstrate that MT, IP, and PD have a substantial and statistically significant influence on the mechanical performance of FDM-printed polymer composite parts. The experimental results, supported by ML-based predictive models, especially those using GPR, confirm that these three parameters play a dominant role in determining tensile and flexural strength. The consistency between experimental and predicted values indicates that the selected factors and their interactions are sufficient to capture the primary process–structure–property relationships within the defined experimental scope. Nevertheless, it is important to recognize the limitations and boundary conditions of the study. All experiments were conducted using a single FDM printer under controlled laboratory conditions with a fixed set of process parameters—specifically, constant layer thickness, nozzle temperature, and printing speed. These parameters were intentionally held constant to isolate the effects of the selected variables. The conclusions drawn from this work are most applicable to settings that closely resemble the conditions used in the study. Variations in printer hardware, filament suppliers, ambient environmental conditions such as temperature and humidity, or adjustments in fixed process parameters may lead to different mechanical outcomes.

Although the present study focused on three key printing parameters—MT, IP, and PD—it is important to acknowledge that other untested parameters may also significantly influence the mechanical properties of FDM-printed parts. Notably, layer thickness and nozzle temperature are widely recognized as critical variables in AM. Layer thickness affects not only the surface finish but also the degree of interlayer bonding. Thinner layers typically promote better layer adhesion due to increased surface contact and more frequent thermal cycling, which can enhance tensile strength and reduce porosity. Thicker layers may reduce printing time but often result in weaker mechanical performance due to reduced bonding area. Similarly, nozzle temperature directly influences the viscosity and flow behavior of the molten filament. Higher nozzle temperatures can improve polymer chain diffusion and interlayer adhesion, particularly in high-performance materials or fiber-reinforced composites. However, excessively high temperatures may lead to material degradation or dimensional inaccuracies. These parameters were held constant in the current study to isolate the effects of the selected variables. Nevertheless, their potential influence on mechanical performance warrants further investigation. Future studies could adopt a more comprehensive DOE approach to include these and other parameters, enabling a more holistic understanding of process–property relationships in FDM.

While recent literature has explored a wide range of advanced ML algorithms—such as XGBoost, random forests, and deep learning-based approaches like CNN-LSTM—for modeling complex mechanical behaviors in AM, this study deliberately focused on two regression models: BLR and GPR. These algorithms were selected based on their suitability for small-to-medium-sized experimental datasets and their ability to provide interpretable predictions with quantified uncertainty. GPR is well-suited for capturing nonlinear relationships in sparse datasets, while BLR offers a probabilistic linear modeling framework that aligns well with the structured nature of the experimental design. Unlike black-box models such as neural networks, these approaches offer greater transparency, which is essential when interpreting the influence of process parameters on mechanical properties. Although we acknowledge the potential of alternative models, our aim was not to conduct an exhaustive benchmarking study but rather to demonstrate the feasibility and effectiveness of integrating ML into a statistically designed FDM optimization framework. Moreover, the study already includes a comparative analysis between two distinct ML models (BLR and GPR) and a classical RSM, which we believe provides a sufficient baseline for evaluating prediction performance. Expanding the model set further was considered beyond the intended scope of this work.

While this approach enhances internal validity, it also limits the generalizability of the findings. For instance, differences in filament quality across manufacturers or fluctuations in environmental conditions during printing could influence interlayer adhesion or dimensional stability, thereby affecting mechanical behavior. Furthermore, hardware-specific characteristics such as extruder design or calibration accuracy may introduce additional variability that was not captured in this study. Building on the current results, future research could extend the parameter space by including additional process variables such as layer thickness, print speed, or nozzle diameter. Similarly, validating the findings across different FDM machines, material suppliers, and environmental conditions would provide further insight into the robustness and transferability of the models. Despite these limitations, the present study offers a focused and well-controlled framework for understanding the mechanical behavior of multi-material and fiber-reinforced structures produced via FDM. The integration of experimental design with ML enables efficient and accurate prediction of mechanical properties, and the methodology presented here can serve as a foundation for broader applications in engineering fields where lightweight, functional components are needed.

Beyond their predictive performance, both GPR and BLR offer the important advantage of providing uncertainty quantification, which is particularly valuable in engineering contexts where safety, reliability, and risk are critical concerns. Unlike traditional regression techniques that yield only point estimates, these Bayesian approaches generate probabilistic outputs, allowing for a more comprehensive interpretation of model predictions. In the case of GPR, the model inherently produces a predictive meaning along with a variance for each prediction, which can be interpreted as a confidence interval. Although no visual uncertainty plots are presented in this study, numerical analysis of the predicted variances indicates that uncertainty remains low across most of the input space, particularly where training data density is high. As expected, slightly increased uncertainty was observed near the edges of parameter space, where extrapolation occurs. This behavior reflects the model’s ability to express lower confidence in less-informed regions, making it a powerful tool for risk-aware decision-making. Similarly, BLR also yields posterior distributions over the predicted outputs by integrating prior assumptions with observed data. The predictive distribution of BLR includes both model uncertainty and noise uncertainty. During testing, the posterior variances computed by the BLR model remained relatively low across all predictions, suggesting that the model was both confident and stable in its estimations. While the uncertainty quantification in BLR is generally less expressive than in GPR—owing to its linear assumptions—it still provides valuable insight into prediction reliability, especially in applications where interpretability and computational simplicity are desired. The ability of both models to provide reliable uncertainty estimates adds an important layer of interpretability and trust to the results. These quantifications can be further leveraged in future studies for robust optimization, reliability-based design, or safety-critical applications in AM processes.

Although GPR has demonstrated strong predictive capabilities in modeling complex, nonlinear relationships in AM, its practical implementation in real-time AM systems presents several challenges. One of the primary limitations is its high computational complexity, which scales cubically with the size of the training dataset (O(n^3^)). This makes GPR computationally expensive and less suitable for real-time applications, especially in industrial environments where large volumes of sensor data are continuously generated. Additionally, GPR requires the inversion of large covariance matrices, leading to high memory usage and slower processing times, particularly on embedded systems or edge devices commonly used in smart manufacturing platforms. Furthermore, standard GPR models are not inherently designed for online or incremental learning, meaning that even minor updates to the dataset may require complete model retraining, posing a bottleneck for adaptive or self-correcting AM systems. Future research could explore the use of sparse GPR approximations, which reduce the model’s complexity by using a subset of the training data, to overcome these limitations. Online GPR algorithms may also offer real-time adaptability by updating the model incrementally as new data becomes available. Moreover, hybrid strategies that combine GPR with faster surrogate models or integrate GPR into cloud-based or parallel computing architectures could allow for real-time feedback without compromising computational efficiency. For successful industrial adoption, a practical roadmap would involve optimizing kernel functions for specific AM processes, developing modular and lightweight software implementations compatible with existing machine controllers, and validating model performance under real-time operating conditions through sensor-integrated experimental setups. These steps would facilitate the transition of GPR from research to production environments, enabling more intelligent and adaptive AM workflows.

## 4. Conclusions

In this study, an extensive experimental and computational investigation was conducted to analyze the effects of key FDM process parameters—MT, IP, and PD—on the tensile and flexural strength of 3D-printed parts. Using a BBD experimental setup, 15 different combinations of printing parameters were tested, and mechanical tests were performed to quantify the resulting strength properties. The statistical significance of each parameter was evaluated through ANOVA, which revealed that MT had the most dominant influence on both tensile and flexural strength, followed by printing direction and infill pattern. Further analysis using Pareto charts and contribution plots confirmed that material selection accounted for over 69% and 76% of the variation in tensile and flexural strength, respectively. This highlights the critical importance of choosing the appropriate filament material—particularly high-performance composites like PPA/Cf—to enhance structural performance. Regression equations were then developed using the BBD approach, resulting in high prediction accuracy, with R^2^ values of 0.9895 for tensile and 0.9885 for flexural strength. These models were validated through normal probability plots and time-series plots, confirming their reliability. To further improve prediction capabilities, ML algorithms, namely GPR and BLR, were implemented in MATLAB using the Regression Learner Toolbox. These models yielded even higher accuracy, with GPR achieving R^2^ values of 0.9935 for tensile strength and 0.9925 for flexural strength. Error performance metrics such as MSE, RMSE, MAE, MAPE, and correlation coefficients demonstrated that GPR consistently outperformed both the BBD and BLR models across all evaluation criteria. Finally, a validation test using unseen sample data confirmed the generalization ability of the models. GPR achieved the lowest MAPE values of 0.54% for tensile strength and 0.45% for flexural strength, proving its robustness and applicability in practical AM scenarios. This study demonstrates that ML algorithms, particularly GPR, can significantly enhance the predictive modeling of mechanical properties in FDM processes. When combined with structured experimental designs like the BBD, such models offer powerful tools for optimizing 3D printing parameters, reducing trial and error, and improving part performance in industrial applications. Future work may involve expanding the dataset, incorporating additional parameters such as layer height or nozzle temperature, and deploying real-time prediction systems in smart manufacturing environments.

## Figures and Tables

**Table 1:** The experimental design’s printing parameters and levels.

Parameters	Symbol	Level 1	Level 2	Level 3
		−1	0	1
Material Type	MT	ABS	PPA/Cf	Sandwich
Infill Pattern	PP	Triangles	Grid	Cross
Printing Direction	PD	Flat	On-Edge	Upright

**Table 2:** The experiments recommended by the BBD.

Run	Printing Parameters		
	Material Type	Infill Pattern	Printing Direction
1	0	0	0
2	−1	0	−1
3	0	1	−1
4	1	−1	0
5	1	0	−1
6	−1	−1	0
7	−1	1	0
8	1	1	0
9	0	−1	1
10	1	0	1
11	−1	0	1
12	0	0	0
13	0	1	1
14	0	0	0
15	0	−1	−1

**Table 3:** The experimental results of the BBD.

Run	Printing Parameters			Output Parameters	
	Material Type	Infill Pattern	Printing Direction	Tensile Strength (MPa)	Flexural Strength (MPa)
1	PPA/Cf	Grid	On-Edge	72.5 ± 1.3	95.8 ± 1.8
2	ABS	Grid	Flat	41.2 ± 1.0	55.4 ± 1.7
3	PPA/Cf	Cross	Flat	75.8 ± 1.1	102.3 ± 2.0
4	Sandwich	Triangles	On-Edge	58.3 ± 1.2	76.2 ± 2.1
5	Sandwich	Grid	Flat	62.1 ± 1.4	84.1 ± 1.5
6	ABS	Triangles	On-Edge	39.6 ± 1.3	51.7 ± 1.9
7	ABS	Cross	On-Edge	43.5 ± 1.2	58.9 ± 1.8
8	Sandwich	Cross	On-Edge	60.2 ± 0.9	80.4 ± 1.3
9	PPA/Cf	Triangles	Upright	62.9 ± 1.1	79.6 ± 1.2
10	Sandwich	Grid	Upright	55.1 ± 1.0	72.3 ± 1.5
11	ABS	Grid	Upright	37.8 ± 1.4	49.5 ± 1.9
12	PPA/Cf	Grid	On-Edge	73.0 ± 1.1	96.1 ± 2.0
13	PPA/Cf	Cross	Upright	67.4 ± 1.3	89.8 ± 1.9
14	PPA/Cf	Grid	On-Edge	72.7 ± 1.0	94.7 ± 2.1
15	PPA/Cf	Triangles	Flat	70.1 ± 1.2	91.5 ± 1.6

**Table 4:** The ANOVA results for the tensile and flexural compressive strength of the parts.

		Tensile Strength				Flexural Strength			
Source	DF	Adj SS	Adj MS	F-Value	*p*-Value	Adj SS	Adj MS	F-Value	*p*-Value
MT	2	605.38	254.69	201.44	0.000	1315.78	658.39	102.23	0.000
IP	2	65.03	36.51	24.87	0.003	141.02	70.51	17.07	0.001
PD	2	195.89	95.94	64.50	0.000	257.13	128.56	31.13	0.000
Error	8	11.51	1.44			33.04	4.13		
Lack of Error	6	11.38	1.90	29.95	0.033	31.96	5.33	9.80	0.095
Pure Error	2	0.13	0.06			1.09	0.54		
Total	14	2463.34				4291.50			

**Table 5:** Prediction results in tensile strength using ML algorithms.

Model	MSE	MAE	RMSE	MAPE (%)	R^2^	Correlation (r)
BBD	0.9015	3.5482	0.9495	13.025	0.9895	0.9947
BLR	0.8711	3.1027	0.9334	12.079	0.9912	0.9956
GPR	0.7933	2.7021	0.8908	11.135	0.9935	0.9967

**Table 6:** Prediction results for flexural strength using ML algorithms.

Model	MSE	MAE	RMSE	MAPE (%)	R^2^	Correlation (r)
BBD	1.2548	3.7524	1.1205	14.102	0.9885	0.9946
BLR	1.2147	3.5875	1.1028	13.692	0.9902	0.9955
GPR	1.1525	3.4219	1.0739	12.958	0.9925	0.9968

**Table 7:** Comparative validation results of models for tensile and flexural strength.

Output Parameters	No.	Printing Parameters			Actual	BBD	GPR	BLR
		MT	IP	PD		Predicted	Predicted	Predicted
Tensile Strength	1	ABS	Cross	Upright	36.81	37.75	36.93	37.34
	2	Sandwich	Triangles	Flat	64.23	63.32	64.68	63.91
	3	PPA/Cf	Cross	Fiat	76.42	75.41	76.87	76.08
Flexural Strength	1	ABS	Triangles	Upright	50.36	51.28	50.56	50.93
	2	Sandwich	Cross	Flat	87.24	86.35	87.68	86.97
	3	PPA/Cf	Triangles	On-Edge	83.57	82.65	83.94	83.28

## Data Availability

The original contributions presented in this study are included in the article. Further inquiries can be directed to the corresponding author.
